# μCT trait analysis reveals morphometric differences between domesticated temperate small grain cereals and their wild relatives

**DOI:** 10.1111/tpj.14312

**Published:** 2019-04-10

**Authors:** Aoife Hughes, Hugo R. Oliveira, Nick Fradgley, Fiona M. K. Corke, James Cockram, John H. Doonan, Candida Nibau

**Affiliations:** ^1^ The National Plant Phenomics Centre Institute of Biological, Rural and Environmental Sciences (IBERS) Aberystwyth University Gogerddan, Aberystwyth SY23 3EE UK; ^2^ School of Earth and Environmental Sciences Manchester Institute of Biotechnology University of Manchester Manchester M1 7DN UK; ^3^ John Bingham Laboratory NIAB Huntingdon Road Cambridge CB3 0LE UK; ^4^ Present address: Computational and Systems Biology and Crop Genetics John Innes Centre Norwich NR4 7 UH UK; ^5^ Present address: Interdisciplinary Center for Archaeology and Evolution of Human Behaviour (ICArEHB) Faculdade das Ciências Humanas e Sociais Universidade do Algarve Campus de Gambelas Faro 8005‐139 Portugal

**Keywords:** X‐ray microcomputed tomography, μCT, wheat, barley, domestication, grain traits, phenomics

## Abstract

Wheat and barley are two of the founder crops domesticated in the Fertile Crescent, and currently represent crops of major economic importance in temperate regions. Due to impacts on yield, quality and end‐use, grain morphometric traits remain an important goal for modern breeding programmes and are believed to have been selected for by human populations. To directly and accurately assess the three‐dimensional (3D) characteristics of grains, we combine X‐ray microcomputed tomography (μCT) imaging techniques with bespoke image analysis tools and mathematical modelling to investigate how grain size and shape vary across wild and domesticated wheat and barley. We find that grain depth and, to a lesser extent, width are major drivers of shape change and that these traits are still relatively plastic in modern bread wheat varieties. Significant changes in grain depth are also observed to be associated with differences in ploidy. Finally, we present a model that can accurately predict the wild or domesticated status of a grain from a given taxa based on the relationship between three morphometric parameters (length, width and depth) and suggest its general applicability to both archaeological identification studies and breeding programmes.

## Significance Statement

Small grain cereals provide the bulk of human calorific intake both directly and indirectly through their use for livestock feed. We present tools based on μCT scanning to morphometrically model differences in grain shape in wild and domesticated wheat and barley. This approach provides insights into past and ongoing changes in grain shape in small grain cereals.

## Introduction

A key factor in the transition from hunting−gathering to settled food production was the cultivation and subsequent domestication of cereals, including wheat and barley (Zeder, 2008; Shewry, 2009). The earliest domesticated forms of wheat were the diploid einkorn, *Triticum monococcum* subsp*. monococcum* (genome AA) and the tetraploid emmer, *T. turgidum* subsp. *dicoccum* (genome AABB), derived from the wild relatives *T. monococcum* subsp. *aegilopoides*, and *T. turgidum* subsp. *dicoccoides*, respectively. Furthermore, domestication of tetraploid wheat eventually gave rise to the modern tetraploid crop, *T. turgidum durum* (pasta wheat) and, through hybridisation with *Aegilops tauschii* (genome DD), to the hexaploid wheats *T. aestivum aestivum* (bread wheat) and *T. aestivum spelta* (spelt). Barley, *Hordeum vulgare*, was also domesticated in the Fertile Crescent from the wild progenitor *H. spontaneum* and, together with wheat, represent the key founder crops on which western agriculture was built (Brown *et al*., 2009; Pankin and von Korff, 2017).

The group of traits that emerged during domestication by human selection, and can distinguish a crop from their wild progenitors, is known as the ‘domestication syndrome’. The term, coined by Hammer (1984), can include morphological, biochemical, developmental and physiological traits (Abbo *et al*., 2014). Some domestication traits are specific to particular ‘domesticated crop‐wild progenitor’ pairs, for example transformation from short and coarse fibres to long and fine ones during cotton domestication (Butterworth *et al*., 2009), or the increased seed capsule size found in domesticated poppies (Zohary *et al*., 2012). Other domestication traits are common across entire groups, such as reduced ear shattering in grasses or indehiscent pods in legumes (Zohary *et al*., 2012). Domestication syndrome traits are often disadvantageous in the wild, and individuals that possess these may succeed only under human management. For example, disarticulation of ears (‘shattering’) in wild grasses increases the area where spikelets might fall and germinate, but non‐shattering variants have arisen in numerous grain crops, presumably because they are easier for farmers to harvest (Doust *et al*., 2014).

Archaeobotanists often infer that the domestication status of cereal remains by establishing indehiscence of the ear or the rachis fragments (Brown *et al*., 2009; Tanno and Willcox, 2012). However, grains are much more frequently found in the archaeological record, especially from the period when agriculture emerged in the Fertile Crescent. In the absence of diagnostic rachis samples, the possibility of distinguishing wild and domesticated cereals based on grain size and shape is a contentious issue that affects discussions on the place(s) and pace of domestication (Zohary *et al*., 2012). From the analysis of archaeological grains, some authors agree that domestication led to an increase in wheat and barley grain size, independently from the evolution of rachis toughness (Nesbitt and Samuel, 1998; Fuller, 2007; Brown *et al*., 2009). Other authors have suggested that the overlap in sizes between archaeological and present‐day wild and domesticated cereal grains indicates that these traits diversified after domestication and a secure diagnosis cannot be made (Abbo *et al*., 2014). Identifying the morphological and genetic basis of domestication syndrome traits is a rich field of research, but it remains an open question whether there are traits that distinguish crops from their wild progenitors that have not yet been described (Stetter *et al*., 2017; Roucou *et al*., 2018).

Other important domestication syndrome traits in cereals include an increase in yield, reduction in seed dormancy, synchronous tillering and more compact growth (Fuller, 2007; Brown *et al*., 2009). Yield itself is a complex trait that can be decomposed into various components, such as seed size and seed number per plant. Increased seed number is mostly achieved by changing seed packing within the inflorescence and by increasing the number of fertile flowers (Preece *et al*., 2017, 2018). Increased seed size is thought to be a major contributor to the increased yield observed in domesticated cereals compared with their wild progenitors, often expressed as a ‘thousand grain weight’ measure (TGW) (Preece *et al*., 2017, 2018). An increase in the duration of growth, resource allocation to reproductive tissues and seed dimensions all contribute to grain size increase. Cultivation methods, such as tillage, are also thought to have contributed to grain size increases as bigger seeds could tolerate sowing at increased depths and therefore be selected for indirectly (Zohary *et al*., 2012). In addition, food processing methods may have led to selection for grains with bigger endosperm as they would be easier and more profitable to mill (Fuh *et al*., 2014). Alternatively, larger grains could have arisen via pleiotropic effects or genetic linkage and driven by selection that favoured larger organs and plants (Kluyver *et al*., 2017). Recently, the effect of grain size on harvestable yield has been noted in water‐limited environments (Rebetzke *et al*., 2016). Grain shape is associated with grain quality, endosperm characteristics and the ability to pack more grains per spikelet (Evers and Millar, 2002). However, in wheat, only a few previous studies have considered changes in grain shape as part of the domestication syndrome (Gegas *et al*., 2010). Consumer preference is also believed to have driven divergent selection in cereal species such as rice (*Oryza sativa*) in which long grain shape is preferred in India, while most Chinese varieties have smaller and rounder grains and recent research has suggested that this might also be true for wheat (Calingacion *et al*., 2014; Liu *et al*., 2016).

Phenotypic variation in wheat grain shape is surprisingly understudied, perhaps due to the difficulty in quantifying this trait using traditional imaging methods. Previous morphometric studies suggest that grain shape does indeed differ between wild and domesticated wheat and barley but they used destructive and laborious methods often focusing on single mapping populations (Giura and Saulescu, 1996; Campbell *et al*., 1999; Dholakia *et al*., 2003; Breseghello and Sorrells, 2007; Sun *et al*., 2009; Gegas *et al*., 2010; Williams and Sorrells, 2014; Bonhomme *et al*., 2016, 2017).

Originally developed as a medical diagnostic tool, X‐ray microcomputed tomography (μCT) is a non‐invasive imaging technique based on differential X‐ray attenuation due to material composition and density. The ability to provide an accurate three‐dimensional (3D) representation and quantification of internal structures in a non‐invasive and non‐destructive way, combined with partial or full automation, means that μCT is a useful tool for studying complex plant morphology. High‐resolution μCT has been successfully used to analyse various plant traits as well as environmental responses (reviewed in Dhondt *et al*., 2010). We have recently shown that using μCT scanning of ripe wheat spikes, combined with an image analysis pipeline, we can accurately extract and measure grain and spike parameters (Strange *et al*., 2015; Hughes *et al*., 2017). In addition to length and width, which can be acquired from photography and flat‐bed scanning, μCT acquires depth, volume and other information that, combined, can provide a more complete description of grain size and shape variation.

In this study, we use μCT analysis to measure differences in cereal grain size and shape between different wild and domesticated cereals. We describe that grain shape changed between domesticated and wild taxa and that the major change was an increase in grain depth. Grain width also increased, but to a lesser extent, while minimal changes in grain length were detected. Grain shape, therefore, seems to be an important component of grain shape changes during domestication. We developed a model able to, for a given species, classify the domestication status of grain samples of a certain taxa. Finally, examination of a panel of 14 diverse European bread wheat varieties, which represent the founders of a diverse multiparent advanced generation intercross mapping population (Cockram and Mackay, 2018), indicates that grain depth remains a variable and plastic trait even in modern cultivars.

## Results

### 3D grain trait feature extraction and analysis

**Figure 1:**
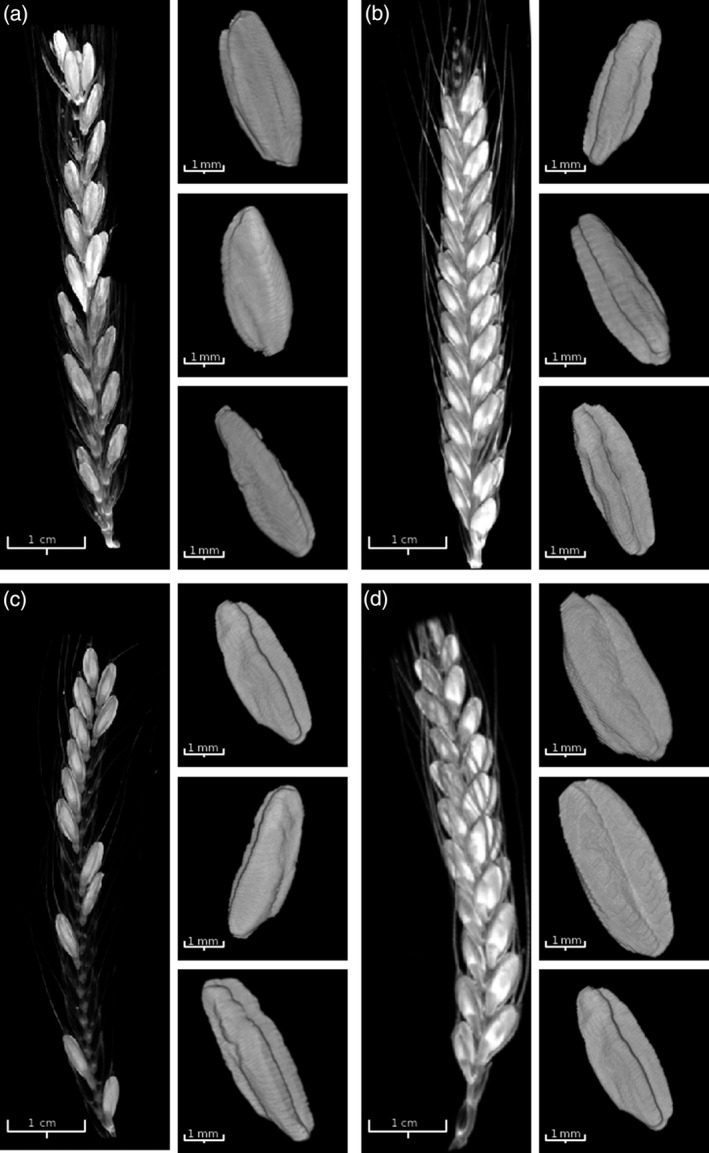
3D visualization of reconstructed μCT images, whole spike (left panels) and representative isolated grains (right panels). (a) wild einkorn, (b) einkorn, (c) wild emmer and (d) emmer. See Movies [Link], [Link], [Link], [Link] for animated gifs of reconstructed spikes.

Several studies have indicated that grain filling, volume and size have undergone changes during domestication (Piperno *et al*., 2004; Gupta *et al*., 2006). To evaluate these morphometric changes we selected four wheat taxa that represent two parallel domestication lines and two different ploidy levels. The diploids, *T. monococcum* subsp. *aegilopoides* (wild einkorn) and *T. monococcum* subsp. *monococcum* (einkorn), and the tetraploids, *T. turgidum* subsp. *dicoccoides* (wild emmer) and *T. turgidum* subsp. *dicoccum* (emmer). Spikes were scanned using μCT (Figure 1 and Movies [Link], [Link], [Link], [Link]) and features extracted using the image analysis software as previously described (Hughes *et al*., 2017). Minor modifications, including an updated pixel distance algorithm and improved grain segmentation, were made to the grain identification algorithms to increase the robustness. A schematic representation of the image analysis pipeline used is provided in Figure S1 and the software is freely available at https://github.com/NPPC-UK/microCT_grain_analyser. The morphological traits measured included grain length, width, depth, volume and surface area.

### Volume differences between wild and domesticated einkorn are mainly due to changes in grain depth

**Figure 2:**
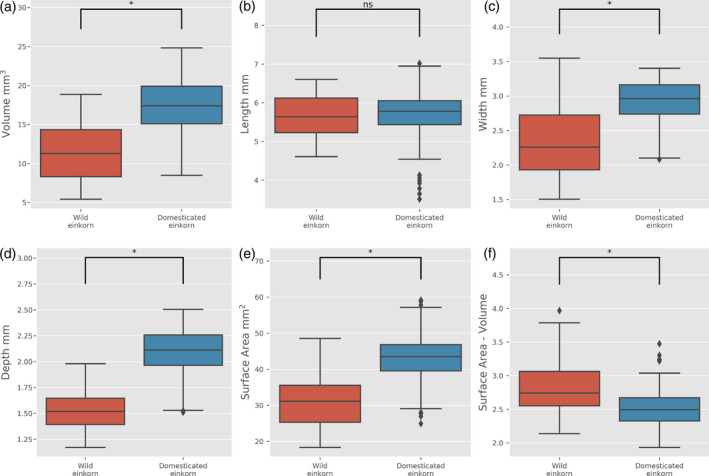
Relationship between domestication status and morphometric trait variation of wild (red boxes, 237 grains) and domesticated (blue boxes, 513 grains) einkorn wheat. Horizontal lines in boxplots represent median, boxes indicate the upper and lower interquartile range, whiskers indicate the largest and smallest values within 1.5 times the interquartile range and points indicate outliers outside this range for grain volume (a), length (b), width (c), depth (d), surface area (e) and surface area to volume ratio (f). Asterisks indicate that the values are significantly different at *P* < 0.01, ns, not significant.

To determine how morphometric traits changed between wild and domesticated einkorn wheat, 237 grains for *Triticum monococcum* subsp. *aegilopoides* (wild) and 513 for *Triticium monococcum* subsp. *monococcum* (domesticated) were analysed and morphological traits measured. We observed a significant increase in grain volume in the domesticated einkorn when compared with wild einkorn (Figure 2a). This finding is in agreement with increases in TGW and two‐dimensional (2D) measurements using the MARVIN grain imager (Figure S2), as well as with previously reported studies (Preece *et al*., 2017). Grain length was not significantly different between the wild and domesticated einkorn (*P* = 0.02) and a predicted 48% probability of overlapping averages by Kruschke's Bayesian estimation of difference (Kruschke, 2013; Figure 2b and Table S1) and this was also observed when we used 2D MARVIN measurements (Figure S2). The major changes between wild and domesticated grains were a small but significant increase in width (Figure 2c), also observed in 2D MARVIN measurements (Figure S2), and a more pronounced increase in depth (Figure 2d). As expected, we observed an increase in surface area (Figure 2e) and a concomitant decrease in the surface area to volume ratio in domesticated einkorn (Figure 2f) as compared with wild einkom.

**Figure 3:**
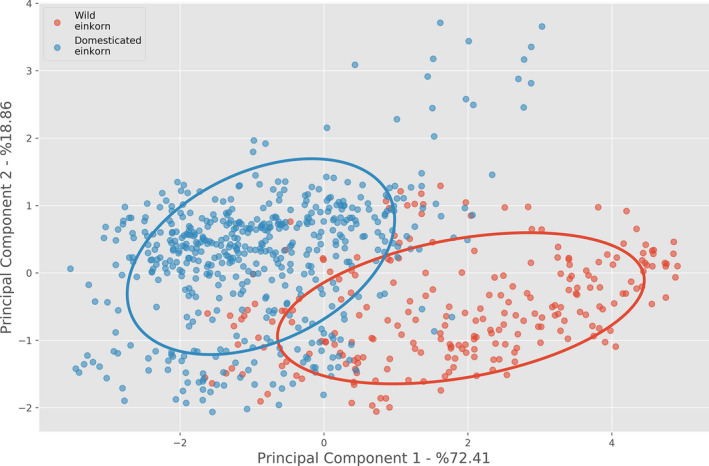
Principal component analysis of grain traits obtained by 3D μCT analysis for wild (red circles, *n* = 237) and domesticated (blue circles, *n* = 513) einkorn wheat, with two times standard deviation outlined. The first two principal components are shown.

In a principal component analysis (PCA), the wild and domesticated groups formed two, slightly overlapping, clusters, with the first principal component (PC) explaining 72% of the variation (Figure 3). Analysis of the loading for each component of PC1 revealed that no dominant trait appeared to influence morphometric variation in einkorn but, as expected, length had the lowest loading value (Table S2). PC2 explained 19% of the variation, with length found to have the main loading value.

### Increased grain depth is also observed between wild and domesticated emmer wheat

**Figure 4:**
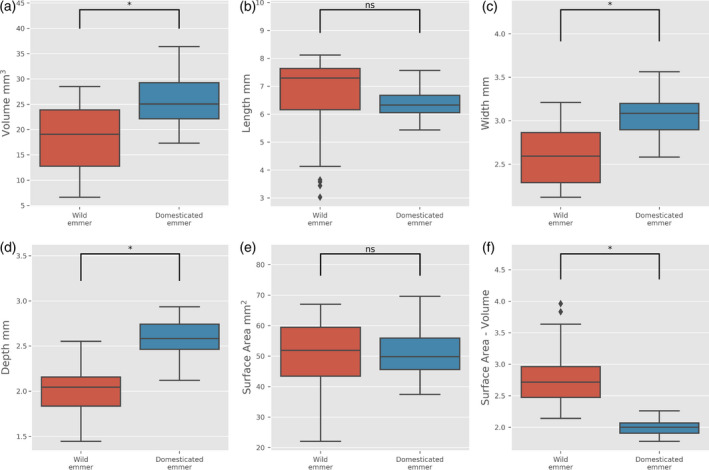
Grain trait analysis of wild (red boxes, 34 grains) and domesticated (blue boxes, 122 grains) emmer wheat. Horizontal lines in boxplots represent the median, boxes indicate the upper and lower interquartile range, whiskers indicate the largest and smallest values within 1.5 times the interquartile range and points indicate outliers outside this range for grain volume (a), length (b), width (c), depth (d), surface area (e) and surface area to volume ratio (f). Asterisks indicate that the values are significantly different at *P* < 0.01, ns, not significant.

As we observed for diploid einkorn wheat, domesticated grains of the tetraploid emmer were associated with a significant increase in grain volume (Figure 4a). This increase can be explained by the increased width and depth of grain, with length not changing significantly (Figure 4b–d). Surprisingly, we did not observe a difference in grain surface area (Figure 4e). This observation was also the case when 2D MARVIN measurements were made (Figure S2), showing that grain shape has significantly altered to preserve this trait value. The observed decrease in the surface area to volume ratio was due to increased grain volume (Figure 4f).

These data suggest that, as with einkorn, changes in grain volume between wild and domesticated grains were the result of changes in size and shape, with the depth component of the grain providing the major effect.

PCA for wild and domesticated emmer grain traits found PC1 and PC2 to explain 72 and 22% of the variation, respectively (Figure S3), finding width, depth, volume and surface area to have the most influence in grain morphology, while length showed the lowest impact. PC2, with much less explanatory power, showed that width and surface area are much less important in explaining variance, while length and depth provide the strongest influence (Figure S3 and Table S2).

### Grain size and shape change across ploidy

**Figure 5:**
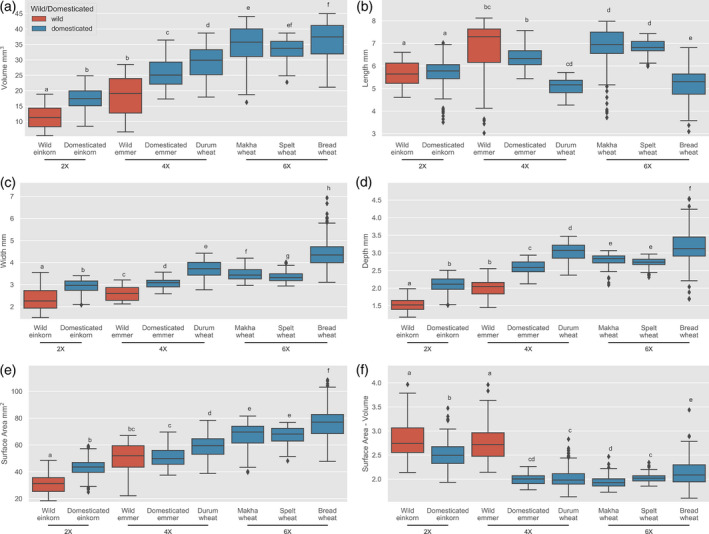
Morphometric changes in wheat grain traits across ploidy and domestication status (wild – red boxes, domesticated – blue boxes). Horizontal lines in boxplots represent median, boxes indicate the upper and lower interquartile range, whiskers indicate the largest and smallest values within 1.5 times the interquartile range and points indicate outliers outside this range for grain volume (a), length (b), width (c), depth (d), surface area (e) and surface area to volume ratio (f). Letters above the boxes indicate significance groups at *P* < 0.01.

To determine the effect of ploidy on grain traits in wheat we extracted grain morphometric features from a variety of wheat taxa differing in their ploidy levels and domestication status. Besides the diploid einkorn and the tetraploid emmer, we also analysed the tetraploid domesticated durum wheat and three domesticated hexaploid wheat, makha wheat, spelt wheat and bread wheat (Figure 5). We found that increased ploidy was associated with increased grain volume, with the domesticated tetraploids being larger than the diploids and the hexaploid than the tetraploids (Figure 5a). As observed with einkorn and emmer, variation in grain length does not generally contribute to the observed volume increases: there was a small but significant increase in length with ploidy increase between einkorn and emmer but this was not the case for durum wheat (Figure 5b). In addition, we observed a small decrease in length for the hexaploid bread wheat. Small changes in grain width are associated with domestication and ploidy (Figure 5c) but the more dramatic change was seen with grain depth (Figure 5d). This observation underscores the effect that grain depth, usually an overlooked trait, has on grain size and shape differences. The surface area to volume ratio was found to have changed more with domestication status than with ploidy, supporting the idea that this trait was under strong selection during crop evolution (Figure 5e).

### Grain shape changes between wild and domesticated barley

**Figure 6:**
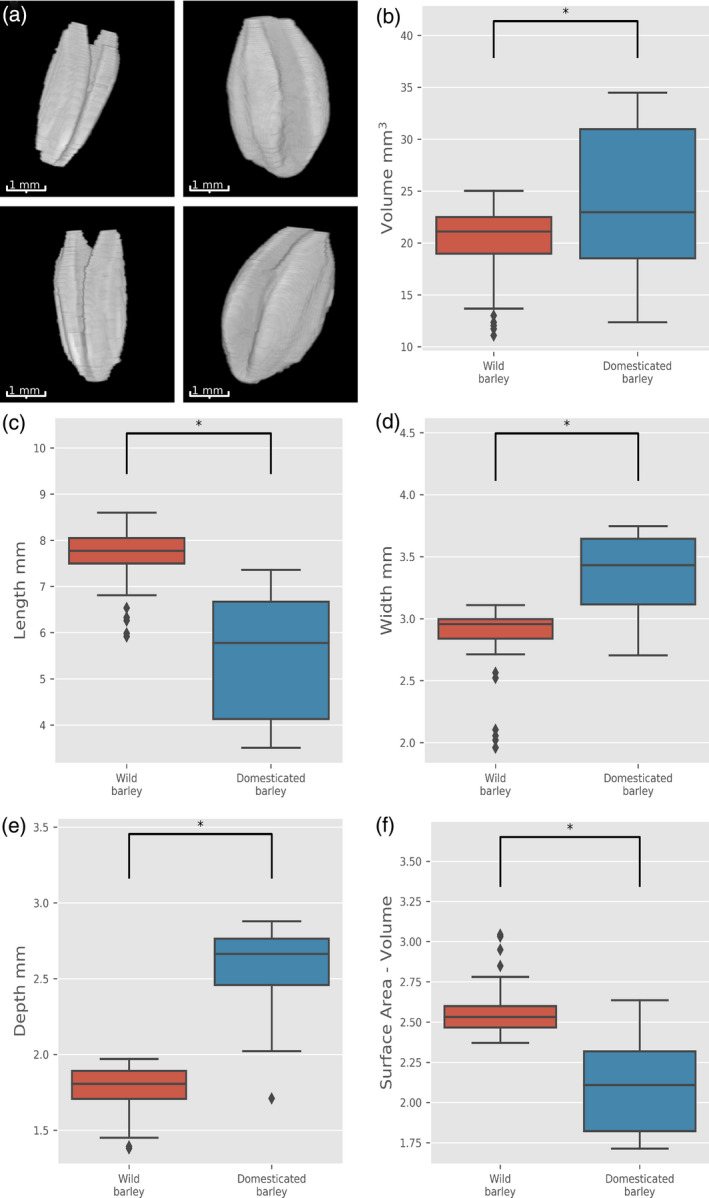
Analysis of grain trait changes associated with barley domestication. (a) 3D reconstruction of representative wild (left) and domesticated (right) barley grains. Grain traits measured were volume (b), length (c), width (d), depth (e) and surface area to volume ratio (f). Horizontal lines in boxplots represent median, boxes indicate the upper and lower interquartile range, whiskers indicate the largest and smallest values within 1.5 times the interquartile range and points indicate outliers outside of this range (red boxes − wild, 49 grains; blue boxes − domesticated, 141 grains). Asterisks indicate that the values are significantly different for *P* < 0.01.

Based on the results described in the sections above, we concluded that size changes between wild and domesticated wheat grain were mostly due to changes in grain depth and, to a smaller extent, in grain width. To determine if this also happened in other Triticeae cereals, we compared grain traits of wild (*H. spontaneum*) and domesticated (*H. vulgare*) 2‐row barley. Figure 6(a) indicates that while grain shape is significantly different between wild and domesticated barley, volume is only marginally changed (Figure 6b). By contrast with that observed for wheat, there was a significant reduction in grain length in domesticated barley, accompanied by increases in grain width and, most notably, in depth (Figure 6c–e). A similar trend was also observed using 2D MARVIN analysis (Figure S2). The surface area to volume ratio decreased as expected in the domesticated grains (Figure 6f).

Taken together, these data suggested that, both in barley and wheat, grain volume increased in an allometric way, with depth and width being responsible for the majority of the volume changes.

### Developing a model to predict domestication in cereals

The morphometric datasets collated here supported the idea that grain size and shape in both wheat and barley changed with domestication status. We next asked if we could quantify the observed changes and develop a mathematical model capable of distinguishing between wild and domesticated grains based on morphological parameters. To this end, we used a multiple regression model to obtain predictions of domestication using three key parameters: grain length, width and depth. This model used an ordinary least squares (OLS) method to estimate weighting of the given features.

**Figure 7:**
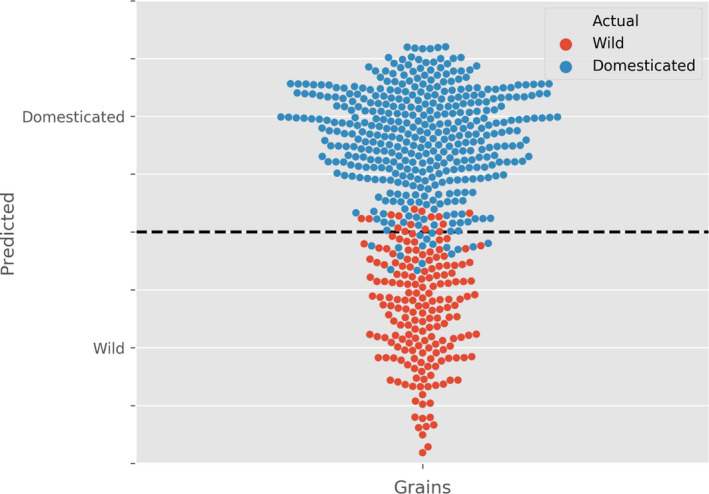
Modelling the domestication status of einkorn wheat grains. Multiple regression model shows the predicted (*y* axis) and actual (wild − red circles and domesticated − blue circles) domestication status of einkorn with an *R* ^2^ value of 0.95 for 750 individual grains analysed.

The OLS model was designed using 20% of the einkorn grain population (a random sample of 750 grains) and then tested against the remaining 80% of the data. Cross‐validation and multiple sampling methods showed that this model is consistently effective at distinguishing domestication status in einkorn, and can predict the domestication status of the grains with high accuracy and a minimal sum of squared errors (*R* ^2^ value of 0.95; Figure 7). A similar level of accuracy was obtained when the model was tested in emmer (total of 125 grains), with an *R* ^2^ value of 0.996 (Figure S4). To test applicability to other cereal grains, we applied the same model to the barley grain dataset (total of 152 grains) and, as observed with wheat, the model was able to predict the domestication status of each grain with high accuracy (*R* ^2^ value of 0.997; Figure S4).

Notably, the grains that the model classified incorrectly were found close to the boundary between the wild and domesticated classes, supporting the suggestion that domestication traits exist in a continuum from wild to domesticated species (Abbo *et al*., 2014) and that such traits generally mapped to quantitative trait loci when mapping populations between wild and domesticated varieties are examined (Yu *et al*., 2018). The accuracy of the model is drastically reduced when depth was not included (reducing the *R* ^2^ value to about 0.3 for all three cereal groups), underscoring the importance of all three dimensions when describing and classifying grain shape traits.

Finally, to estimate if the model was robust to different growth conditions, we re‐grew a population of wild and domesticated einkorn wheat under controlled conditions. Domesticated einkorn varieties reached key growth stages GS39 (flag leaf ligule just visible), GS55 (ear half emerged) and GS65 (anthesis half way) earlier when compared with the wild varieties (Figure S5). The hexaploid bread wheat elite variety Paragon, underwent development even more rapidly. Total ear weight and harvest index, two key indicators of domestication and crop yield, were also higher in the domesticated einkorn and the elite bread wheat (Figure S5). Fully mature and dried primary spikes were scanned using μCT and grain parameters extracted (Figure S6) as described above, and were found to show the same trends between wild and domesticated as observed with the einkorn obtained from the stock centres and shown in Figure 2 above. When the model was applied to this new dataset, we again found that it could accurately classify wild and domesticated grains with a *R* ^2^ value of 0.964 (Figure S4). In addition, as was the case for the other datasets, accuracy was notably decreased if depth was not included (*R* ^2^ value of 0.39). These data showed that, within each taxa, morphometric traits can be used to accurately describe and classify domesticated grains.

Taken together our data showed that grain shape and size have changed between wild and domesticated wheat as well as barley and that the observed differences were due in part to changes in grain depth. In addition, we have developed a model that accurately predicted whether a grain of a certain taxa is wild or domesticated. The accuracy of the model depended on all three dimension parameters when comparing grain size and shape across ploidy and domestication events.

## Discussion

Crop domestication has attracted much speculation over the years, and has recently attracted renewed attention because a range of experimental approaches now allow hypotheses to be directly addressed. These ranged from sophisticated analyses of archaeobotanical data, indicating that domestication is a dynamic process that occurred independently in various regions and involved many different crops (Fuller, 2007), to the *de novo* domestication of wild crop‐relatives using genome editing (Lemmon *et al*., 2018; Li *et al*., 2018; Zsögön *et al*., 2018). These new developments mean that improved understanding of crop domestication could have direct implications for how we deal with human population pressure and climate change. As crops became more dependent on humans and a very few species became favoured (Fuller, 2007), the diversity of utilised plant species decreased. It is now possible to conceive that this bottleneck could be reversed, but to help do so we need to understand how traits have changed between domesticated plants and their wild relatives in a lot more detail.

### Depth as a key driver for grain shape changes during domestication

One particular domestication‐related change, observed across many cereal species, is an increase in grain volume. This study shows that the change in volume can be accounted for by a simple morphometric model in which shape changes allometrically, with grain depth being the major explanatory factor. Some studies have previously suggested that grain size and shape have changed during domestication but, due to the nature of the methods used, the 3D shape of the grains was often overlooked (Peng *et al*., 2003; Fuller, 2007; Gegas *et al*., 2010; Okamoto *et al*., 2012; Williams and Sorrells, 2014; Kumar *et al*., 2016; Bonhomme *et al*., 2017; Qin *et al*., 2017). The methodology presented here provides a new tool to now address this directly.

While it has been suggested that grain length changed during domestication (Fuller, 2007; Gegas *et al*., 2010; Okamoto *et al*., 2012), in our data, grain length was not affected by the domestication status of either einkorn or emmer. This difference could be dependent on the type and size of the land race accessions and varieties used or the measuring methods (in our case the hull portion is removed by the segmentation algorithm and not measured). In barley, we observed a small but significant decrease in grain length in domesticated barley samples relative to *H. spontaneum*.

### Possible molecular and developmental mechanisms underlying grain shape variation

The observed grain shape changes could have been brought about by changes in the organisation and size of the different components in the floret. Although it is difficult to distinguish if the changes in grain size are a direct consequence of changes in floral organ size, or just part of the pleotropic effect of increased cell size in the grain, some recent studies have establish a relationship between floral organ size and grain size and shape. In rice, it has been proposed that changes in grain width are controlled by the effect of *GW2* on the hull and by *GW5* that regulates the size of the outer glume (Song *et al*., 2007; Weng *et al*., 2008; Ma *et al*., 2016). *GW2* homologues have been identified in wheat, and gene silencing and mutation studies have suggested that they have a negative effect on grain size by regulating pericarp cell size and length (Zhang *et al*., 2013; Simmonds *et al*., 2016; Qin *et al*., 2017). It will be interesting to determine if other genes that control grain yield in wheat such as *Q*,* S* and *P1* (Salina *et al*., 2000; Sourdille *et al*., 2000; Okamoto and Takumi, 2013) also lead to changes in floret component size and changes in grain shape. We speculate that floret structure was also under selection from human cultivation, so that the size and position of the outer organs increased to allow for larger grains. For example, the position of the lemma and palea may restrict increased grain depth. Interestingly, a quantitative trait locus (QTL) controlling grain shape was recently identified in tetraploid wheat and overlays the *tenacious glume* locus that controls the toughness of the glume and lemma and that might constrain grain expansion in width and depth (Nalam *et al*., 2007; Okamoto *et al*., 2012).

### The effect of polyploidization on grain morphometric traits

Both einkorn and emmer wheat were domesticated in the Fertile Crescent and, while both species may have faced similar environmental and human selection pressures, a number of grain trait differences have emerged. Some of these differences are likely to have been attributed to the difference in ploidy between the two species. It is generally accepted that increased ploidy results in bigger cells, with examples of increased organ size associated with ploidy observed in both plant and animal species (Orr‐Weaver, 2015). In agreement with this observation, grain volume and surface area both increase with increased ploidy. However, grain length does not change markedly with ploidy, and indeed decreases slightly in durum and bread wheat. Recent studies on the function of *TaGW2*, a key determinant of grain weight in wheat, suggested that grain size increase, especially in width, mainly occurred from the diploid to tetraploid transition associated with decreased genetic diversity of *TaGW2*. Interestingly, comparison of polymorphisms and genetic diversity of *TaGW2* across ploidies and domestication status as well as *TaGW2* expression, suggested that polyploidization events possibly caused much stronger differentiation than domestication and breeding (Qin *et al*., 2017). Opposing results of increased and reduced grain size upon downregulation or loss of *TaGW2* (Bednarek *et al*., 2012; Zhang *et al*., 2018) indicated a complex interaction between several genes within a specific genetic background and that the effect of ploidy on the function and selection process of the genes underlying grain traits is complex.

### Agro‐ecological significance of larger grains

Even though some authors suggested that increased grain size was one of the earliest traits to have developed during cereal domestication (Nesbitt and Samuel, 1998; Brown *et al*., 2009), it is unlikely that this trait developed in isolation, or for the sole purpose of producing larger grains. In open environments in which agriculture began, larger seeds would have the significant advantage by being able to survive deeper burial due to their increased ability to absorb water and nutrients and generally produce larger seedlings that emerged faster, or they might have resulted from indirect selection of bigger plants with generally bigger organs (Chastain *et al*., 1995; Fuller, 2007; Kluyver *et al*., 2013). Although grain shape has not traditionally been a direct breeding target in wheat and barley, our data suggest that it is critical when describing taxa‐specific changes in grain characteristics from wild to domesticated forms. Grain depth, as a major driver in grain shape changes, could be an important target for increasing productivity. Notably, we observe significant variation in grain depth within modern wheat cultivars (Figure S7), suggesting that this trait could be a target in breeding programmes.

### Modelling grain domestication

Several modelling studies point to the importance of grain shape in breeding varieties with better yield and milling properties, suggesting that large spherical grains would be ideal (Evers and Millar, 2002; Fuh *et al*., 2014). In the wild and domesticated pairs we analysed, in parallel to the expected increase in volume, there was a clear change in grain shape towards more spherical grains. A good descriptor of this change is the ratio between grain surface area (SA) and volume. Although it has economic implications for the milling industry, in which a bigger endosperm to bran ratio is highly desirable (Evers and Millar, 2002), obtaining accurate measurements are still challenging. Using the 3D μCT approach, both SA and volume can be accurately calculated. For einkorn, emmer and barley we found a significant decrease in the SA/volume ratio with the domestication status of the grain. Changes in seed shape associated with domestication have been previously reported in other plant species, and serve as diagnostic features to distinguish true wild populations from cultivated or feral populations (Terral *et al*., 2010; Gros‐Balthazard *et al*., 2016), but this has not been reported for cereals. Taking into account the relationship between the three grain dimensions, length, width and depth, a multiple regression model was generated that allowed the accurate classification of grains of a given taxa as wild or domesticated. The model also provided further evidence of the importance of grain depth when analysing changes in grain size and shape. For example, when depth was included in the einkorn model, domestication status was predicted with an *R* ^2^ of 0.95 while, if only length and width were considered, the *R* ^2^ decreased to 0.31. Similar differences were observed for all three cereal pairs tested. Notably, the grains that the model classifies incorrectly lay in the edges of the two classifications, reflecting the quantitative nature of grain morphometric traits.

Grain identification (taxa and domestication status) from archaeological sites can provide information on associated agricultural practices, on the evolution of nutrition and about possible migration routes and other cultural aspects (Jacomet, 2006). Poorly preserved grain may preclude DNA analyses, so these inferences are drawn on morphological traits and, while increased grain volume is often used in the identification of domesticated grain, it is quite difficult to measure accurately. For some taxa such as barley, there was a relatively small change in volume associated with domestication (Willcox, 2004; Fuller, 2007), making it difficult to distinguish domesticated grain from that of wild relatives. The methodology and modelling based on 3D analysis of grains developed here represents a useful tool for the identification of the domestication status of a grain for a given taxa. Further work applying this approach to archaeological materials, and building on work done with charred material (Bonhomme *et al*., 2017), could help to elucidate the diversity of forms that existed at the onset of agriculture and subsequent millennia. Re‐analysis of archaeobotanical remains from key Fertile Crescent archaeological sites using these techniques may help to resolve old debates about the ‘when’ and ‘where’ of the origins of agriculture.

## Experimental procedures

### Plant materials

The wheat taxa used in this study were the diploids *T. monococcum* subsp. *aegilopoides* (wild einkorn) and *T. monococcum* subsp. *monococcum* (einkorn), the tetraploids *T. turgidum* subsp. *dicoccoides* (wild emmer), *T. turgidum* subsp. *dicoccum* (emmer) and *T. turgidum* subsp*. durum* (domesticated durum wheat) and the hexaploid *T. aestivum* subsp*. macha* (domesticated Makha wheat), *T. aestivum spelta* (domesticated spelt wheat) and *T. aestivum* subsp. *aestivum* (domesticated bread wheat). Einkorn spikes for scanning were obtained from the USDA (GRIN) and emmer spikes from the John Innes Centre collection and encompass a wide range of habitats of origin (Table S3). Spikes from *T. aestivum* subsp*. macha* and *T. aestivum spelta* were obtained from the John Innes Centre Germplasm Resource Unit (Table S3) and spikes for *T. turgidum* subsp*. durum* and *T. aestivum* subsp. *aestivum* were present at the National Plant Phenomics Centre (NPCC), Aberystwyth, UK. For barley, examples of the wild (*H. spontaneum*) and the domesticated 2‐row (*H. vulgare*) species were obtained from the USDA (GRIN) and grown at the NPPC and mature fully dried spikes were selected. This was also the case for the test einkorn population. The 14 bread wheat varieties used were Banco, Bersee, Brigadier, Copain, Cordiale, Flamingo, Kolka, Maris Fundin, Robigus, Slejpner, Soissons, Spark, Steadfast and Stetson (Cockram and Mackay, 2018), with ears sourced from field trials at NIAB, Cambridge. Bread wheat trials were conducted in the 2015–2016 growing season when ears were randomly selected from plots measuring 1 × 1.2 m. For the glasshouse experiments (both barley and test einkorn populations), seed was sown in 6 cm pots of F2 compost (Levington, UK) grown for 2 weeks (18/15°C, 14 h day length) before transfer for 6 weeks vernalisation (5°C, 16 h day length). Post vernalisation plants were re‐potted into 3.5 L pots of the same compost and grown with uniform watering and additional lighting in the glasshouse until harvest (18/15°C 14 h day length). Plants were grown during the autumn/winter of 2016 and growth stages scored according to the Zadoks scale (Zadoks *et al*., 1974) and the days taken to reach GS39 (flag leaf ligule just visible), GS55 (ear half emerged) and GS65 (anthesis half way) from the sowing date noted. Once fully ripened, the total weight of all the ears of the plant and the harvest index (calculated by dividing the total ear weight by the total plant weight) was determined.

Two‐dimensional grain measurements for length, width, area and estimated TGW were performed on more than 50 grains per genotype using a MARVIN grain analyser (GTA Sensorik GmbH, Germany).

### 3D scanning of spikes

From the genotypes selected, fully dried, representative spikes were chosen for μCT scanning. Spikes were placed in plastic holders (34 × 70 mm tubes) and imaged using a μCT100 scanner (Scanco Medical, Switzerland). Spikes were scanned with the X‐ray power set at 45 kVp, 200 μA and 9 W with an integration time of 200 msec. Each spike was ~1000 slices (51 slices per stack), 125 projections/180° were taken and a binning of 6 was used. Output images were produced with a 0.2 megapixel (512 × 512) resolution (68.8 μm/pixel) in a proprietary ISQ file type format (Scanco Medical, Switzerland).

### Morphometric feature extraction

Feature extraction was performed using previously developed MATLAB‐based software (Hughes *et al*., 2017) and freely available at https://github.com/NPPC-UK/microCT_grain_analyser. Changes were made to the existing pipeline for the watershedding and segmentation processes, to work with the wild wheat taxa (Figure S1). The features extracted were length (calculated using the major axis of the whole grain), width and depth (the major and minor axis of a cross section respectively, found by selecting the grain's midpoint), volume (a complete connected pixel count per grain) and surface area (single pixel perimeter calculation mapped in three dimensions). The data were checked for false positives by first removing outliers that were identified using the 0.025 upper and lower percentiles of the data. Additionally, for added robustness, constraints of expected grain limits were applied to the data based on findings from previous studies (Hughes *et al*., 2017).

All the μCT scan files and the data analysis files are available at the following https://doi.org/10.20391/d25bda3e-8725-485c-a4e8-fa61f0096eaa.

### PCA and Bayesian modelling and significance testing

To provide deeper insight into the size of change or similarity in hypothesis testing, a Bayesian model was used. To estimate the probability of two samples containing the same mean, the method used Bayes theorem along with Markov‐Chain−Monte‐Carlo (MCMC) to draw random samples from a normal population (Kruschke, 2013). To quantify differences in population means, a percentage likelihood of difference was produced using Kruschke's method. PCA was performed using the python toolkits ‘scikit‐learn’ (Pedregosa *et al*., 2011) and the ‘CT Analysing Library’ (Hughes, 2019). Significance analysis was performed using the python library ‘scipy’ (Jones *et al*., 2001) implementation of the Welch test for significance. Shapiro–Wilk tests were used to test data normality and shape.

### Multiple regression modelling

Each model (einkorn, emmer, barley and test einkorn) was trained using 20% of the given data and 80% was used to test and calculate an *R* ^2^ value. Estimation of domestication status was achieved through multiple regression, using OLS methods. Three variables were chosen to define the model: width, length and depth. These variables provided respective coefficients (beta), which formed the basis of the regression model according to the following equation:

$$Y=\beta_{0}\text{length}\times \beta_{1}\text{depth}\times \beta_{2}\text{width}+\epsilon$$

The model was built using the ‘sklearn’ (Pedregosa *et al*., 2011) and ‘statsmodels’ python libraries, and data prepared using the ‘sklearn standard scaler’. Data were split using the ‘sklearn train test split’ function. ‘Statsmodels formula api’ was used to create the regression formula. Finally, ‘statsmodels OLS api’ was used to run, train and test the model. An *R* ^2^ value was produced using the sum of data variance over the explained sum of squares:

$$1-\frac{\sum_{i=0}^{n}r^{2}}{\sum_{i=0}^{n}{\left(y_{i}-\overset{¯}{y}\right)}^{2}}$$

## Author contributions

CN, JHD, JC and HRO conceived the project and designed the experiments. FC and NF grew and manually measured and scored the plants/grain. CN performed the μCT scanning. AH developed the μCT imaging analysis and feature extraction and data analysis pipelines. AH, HRO and CN analysed the data. AH, HRO and CN wrote the manuscript. CN and JHD oversaw the data analysis. JHD and JC obtained funding. All authors edited and approved the final manuscript.

## Conflict of interest

The authors declare no conflict of interest.

## Supporting information


**Figure S1**. Schematics of the μCT scanning, image analysis and feature extraction pipeline used in this study.
**Figure S2**. 2D analysis of grain length, width, area and predicted thousand grain weight (TGW) of the indicated taxa using MARVIN imaging.
**Figure S3**. Principal component analysis of grain traits obtained by 3D μCT analysis for wild (red circles) and domesticated (blue circles) emmer wheat.
**Figure S4**. Modelling of the domestication status of emmer, barley and einkorn grains.
**Figure S5.** Analysis of domestication‐related traits in wild (five plants) and domesticated (20 plants) einkorn wheat when compared with the modern hexaploid bread wheat variety Paragon (five plants).
**Figure S6**. Analysis of grain traits in wild (red boxes) and domesticated (blue boxes) einkorn wheat population grown at the NPPC.
**Figure S7**. Grain depth analysis of 14 hexaploid wheat varieties.

**Table S1.** Significance testing and Bayesian likelihood of similarity for the different populations used.
**Table S2.** Loading values for principal component 1 (PC1) and principal component 2 (PC2) for the einkorn and emmer principal component analyses (PCAs) shown in Figures 3 and S3, respectively.
**Table S3.** Taxa, common names and collection accession numbers for the lines used in this study.

**Movie S1.** Representative ear of partially reconstructed wild einkorn wheat.

**Movie S2.** Representative ear of einkorn wheat.

**Movie S3.** Representative ear of partially reconstructed wild emmer wheat.

**Movie S4.** Representative ear of emmer wheat.
