# Supplementary material for: μCT trait analysis reveals morphometric differences between domesticated temperate small grain cereals and their wild relatives
Source: Plant J. 2019 Apr 10;99(1):98–111. doi: 10.1111/tpj.14312 (PMC6618119; doi:10.1111/tpj.14312)
Supplement: Supplementary file 2 — Table S1. Significance testing and Bayesian likelihood of similarity for the different populations used. Table S2. Loading values for principal component 1 (PC1) and principal component 2 (PC2) for the einkorn and emmer principal component analyses (PCAs) shown in Figures 3 and S3, respectively. Table S3. Taxa, common names and collection accession numbers for the lines used in this study. [file TPJ-99-98-s006.docx]

**SUPPORTING INFORMATION**

**SUPPLEMENTAL TABLES**

**Supplemental Table 1.** Significance testing and Bayesian likelihood of similarity for the different populations used.

| **Einkorn** | **p-val** | **t-val** | **diff_ mean** | **0.025** | **0.975** | **Bayesian likelihood** |
| --- | --- | --- | --- | --- | --- | --- |
| **volume** | <0.001 | 22.4 | 5.98 | 5.48 | 6.49 | 0.01 |
| **length** | 0.028 | 2.20 | 0.09 | 0.01 | 0.18 | 0.48 |
| **width** | <0.001 | 15.1 | 0.57 | 0.51 | 0.63 | 0.11 |
| **depth** | <0.001 | 35.7 | 0.56 | 0.53 | 0.59 | 0.06 |
| **surface area** | <0.001 | 22.3 | 11.91 | 10.95 | 12.87 | 0.04 |

| **Emmer** | **p-val** | **t-val** | **diff_ mean** | **0.025** | **0.975** | **Bayesian likelihood** |
| --- | --- | --- | --- | --- | --- | --- |
| **Volume** | <0.001 | 5.81 | 7.43 | 5.39 | 9.48 | 0.25 |
| **length** | 0.539 | -0.62 | -0.17 | -0.49 | 0.14 | 0.50 |
| **width** | <0.001 | 7.35 | 0.47 | 0.37 | 0.57 | 0.42 |
| **depth** | <0.001 | 11.3 | 0.58 | 0.50 | 0.66 | 0.36 |
| **surface area** | 0.363 | 0.92 | 2.25 | -1.15 | 5.64 | 0.44 |

| **Barley** | **p-val** | **t-val** | **diff_ mean** | **0.025** | **0.975** | **Bayesian likelihood** |
| --- | --- | --- | --- | --- | --- | --- |
| **Volume** | <0.001 | -5.67 | -4.44 | -6.44 | -2.45 | 0.34 |
| **length** | <0.001 | 15.6 | 2.22 | 1.84 | 2.60 | 0.20 |
| **width** | <0.001 | -10.8 | -0.54 | -0.63 | -0.44 | 0.37 |
| **depth** | <0.001 | -28.0 | -0.82 | -0.89 | -0.75 | 0.23 |
| **surface area** | 0.315 | 1.01 | 1.34 | -1.36 | 4.03 | 0.47 |

| **Test Einkorn** | **p-val** | **t-val** | **diff_ mean** | **0.025** | **0.975** | **Bayesian likelihood** |
| --- | --- | --- | --- | --- | --- | --- |
| **Volume** | <0.001 | 12.9 | 5.38 | 4.53 | 6.23 | 0.24 |
| **length** | 0.014 | 2.48 | 0.13 | 0.03 | 0.23 | 0.50 |
| **width** | <0.001 | 8.86 | 0.41 | 0.32 | 0.50 | 0.38 |
| **depth** | <0.001 | 14.8 | 0.38 | 0.33 | 0.44 | 0.38 |
| **surface area** | <0.001 | 11.1 | 9.89 | 8.07 | 11.70 | 0.29 |

| **Ploidy Wild** | **p-val** | **t-val** | **diff_ mean** | **0.025** | **0.975** | **Bayesian likelihood** |
| --- | --- | --- | --- | --- | --- | --- |
| **volume** | <0.001 | -5.62 | -6.91 | -8.40 | -5.41 | 0.21 |
| **length** | <0.001 | -3.10 | -0.87 | -1.13 | -0.60 | 0.45 |
| **width** | 0.002 | -3.31 | -0.23 | -0.43 | -0.03 | 0.42 |
| **depth** | <0.001 | -9.50 | -0.48 | -0.56 | -0.41 | 0.33 |
| **surface area** | <0.001 | -7.31 | -17.54 | -20.58 | -14.51 | 0.21 |

| **Ploidy domesticated** | **p-val** | **t-val** | **diff_ mean** | **0.025** | **0.975** | **Bayesian likelihood** |
| --- | --- | --- | --- | --- | --- | --- |
| **Volume** | <0.001 | -18.53 | -8.36 | -9.05 | -7.66 | 0.06 |
| **length** | <0.001 | -12.4 | -0.60 | -0.71 | -0.49 | 0.38 |
| **width** | <0.001 | -5.31 | -0.13 | -0.18 | -0.07 | 0.43 |
| **depth** | <0.001 | -27.2 | -0.50 | -0.55 | -0.46 | 0.23 |
| **surface area** | <0.001 | -11.4 | -7.88 | -9.06 | -6.69 | 0.27 |

**Supplemental Table 2.** Loading values for principle component 1 (PC1) and principal component 2 (PC2) for the einkorn and emmer Principle Component Analyses (PCAs) shown in Figure 3 and Figure S3, respectively.

| Einkorn |  |  |  | Emmer |  |  |
| --- | --- | --- | --- | --- | --- | --- |
|  | **PC1** | **PC2** |  |  | **PC1** | **PC2** |
| **width** | -0.41 | 0.11 |  | **width** | -0.42 | -0.30 |
| **length** | -0.24 | -0.87 |  | **length** | -0.31 | 0.72 |
| **depth** | -0.38 | 0.47 |  | **depth** | -0.39 | -0.49 |
| **volume** | -0.46 | -0.04 |  | **volume** | -0.46 | -0.05 |
| **surface area** | -0.45 | -0.06 |  | **surface area** | -0.40 | 0.37 |

**Supplemental Table 3.** Scientific name, common names and collection accession numbers for the lines used in this study.

| **Scientific name** | **Common name** | **Accession numbers used** |
| --- | --- | --- |
| *Triticum monococcum* subsp. *aegilopoides* | wild einkorn | PI427923, PI427495 |
| *Triticum monococcum* subsp. *monococcum* | einkorn | PI167611, PI307984, PI427927, PI94743 |
| *Triticum turgidum* subsp. *dicoccoides* | wild emmer | T106007, T106009, T106010 |
| *Triticum turgidum* subsp. *dicoccum* | emmer | T107001 |
| *Triticum aestivum* subsp. *macha* | makha wheat | T1240001 |
| *Triticum aestivum* subsp. *spelta* | spelt | T1220017 |
| *Hordeum spontaneum* | wild barley | PI227301, PI531853 |
| *Hordeum vulgare* | barley | PI289811, PI327704, PI572573 |
