# Supplementary material for: μCT trait analysis reveals morphometric differences between domesticated temperate small grain cereals and their wild relatives
Source: Plant J. 2019 Apr 10;99(1):98–111. doi: 10.1111/tpj.14312 (PMC6618119; doi:10.1111/tpj.14312)
Supplement: Supplementary file 7 [file TPJ-99-98-s002.docx]

**FULL SUPPORTING INFORMATION LEGENDS**

**Figure S1.** Schematics of the µCT scanning, image analysis and feature extraction pipeline used in this study.

**Figure S2.** 2D analysis of grain length, width, area and predicted thousand grain weight (TGW) of the indicated cereals using MARVIN 2D imaging. Bars represent average ± SD of more than fifty grains.

**Figure S3.** Principle Component Analysis of grain traits obtained by 3D µCT analysis for wild (red circles) and domesticated (blue circles) emmer wheat. Two times standard deviation is represented by the outlined area.

**Figure S4.** Modelling of the domestication status of emmer, barley and einkorn grains. Multiple regression model shows the predicted (y axis) and actual (wild- red circles and domesticated- blue circles) domestication status for each grain.  R^2^ values and accuracy (Acc) for emmer (125 grains) barley (152 grains) and for the locally grown einkorn (148 grains) are shown above each panel.

**Figure S5.** Analysis of domestication-related traits in wild (5 plants) and domesticated (20 plants) einkorn wheat when compared with the modern hexaploid bread wheat variety Paragon (5 plants). (A)- Number of days until growth stage 39 (GS39, flag leaf ligule just visible), GS55 (ear half emerged) and GS65 (anthesis half way). (B)- Total ear weight at maturity. (C)- Harvest index for each taxa calculated as the total ear weight divided by the total plant weight. Bars represent averages ± SE and asterisks indicate that the values are significantly different from the wild variety at p<0.01.

**Figure S6.** Analysis of grain traits in wild (red boxes) and domesticated (blue boxes) einkorn wheat population grown at the NPPC. Horizontal lines in boxplots represent median, boxes indicate the upper and lower interquartile range, whiskers indicate the largest and smallest values within 1.5 times the interquartile range and points indicate outliers outside of this range for grain volume (A), length (B), width (C), depth (D), surface area (E) and surface area to volume ratio (F). Asterisks indicate that the values are significantly different at p<0.01. ns- not significant.

**Figure S7.** Grain depth analysis of 14 hexaploid wheat elite cultivars. Horizontal lines in boxplots represent median, boxes indicate the upper and lower interquartile range, whiskers indicate the largest and smallest values within 1.5 times the interquartile range and points indicate outliers outside of this range. Letters above the boxes significance groups where varieties with the same letter do not differ significantly at 95% confidence intervals p<0.05 (including the Bonferroni correction method for multiple comparisons).

**Table S1.** Significance testing and Bayesian likelihood of similarity for the different populations used.

**Table S2.** Loading values for principle component 1 (PC1) and principal component 2 (PC2) for the einkorn and emmer Principle Component Analyses (PCAs) shown in Figure 3 and Figure S3, respectively.

**Table S3.** Taxa, common names and collection accession numbers for the lines used in this study.

**Movie S1.** Representative ear of partially reconstructed wild einkorn wheat.

**Movie S2.** Representative ear of einkorn wheat.

**Movie S3.** Representative ear of partially reconstructed wild emmer wheat.

**Movie S4.** Representative ear of emmer wheat.
